# Supplementary material for: Plasticity across levels: Relating epigenomic, transcriptomic, and phenotypic responses to osmotic stress in a halotolerant microalga
Source: Mol Ecol. 2022 Jun 9;31(18):4672–87. doi: 10.1111/mec.16542 (PMC9543585; doi:10.1111/mec.16542)
Supplement: Supplementary file 1 — TABLE S1 Mapping statistics for RNA‐seq and WGB‐seq data. TABLE S2 Effects of salinity or initial density on population growth rate for CCAP 19/12 and 19/15. TABLE S3 Strain and salinity effects on population growth rate for isogenic populations. FIGURE S1 Genetic variation within and among populations. FIGURE S2 Dynamics of isogenic populations under different osmotic stresses FIGURE S3 Gene ontology (GO) term enrichment analysis. [file MEC-31-4672-s001.docx]

**Plasticity across levels: relating epigenomic, transcriptomic, and phenotypic responses to osmotic stress in a halotolerant microalga**

Christelle Leung^1*^, Daphné Grulois^1^ and Luis-Miguel Chevin^1^

^1^CEFE, Université de Montpellier, CNRS, EPHE, IRD, Montpellier, France

^*^Correspondance: Christelle Leung : [christelle.leung@umontreal.ca](mailto:christelle.leung@umontreal.ca)

# Supplementary Information

Supplementary information include:

- Table S1. Mapping statistics for RNA-seq and WGB-seq data.
- Table S2. Effects of salinity or initial density on population growth rate for CCAP 19/12 and 19/15.
- Table S3. Strain and salinity effects on population growth rate for isogenic populations.
- Fig. S1. Genetic variation within and among populations.
- Fig. S2. Dynamics of isogenic populations under different osmotic stresses.
- Fig. S3. Gene ontology (GO) term enrichment analysis.

**Table S1. Mapping statistics for RNA-seq and WGB-seq data.**

| **BioSample ID** | | SAMN19677492 | SAMN19677495 | SAMN19677496 | SAMN19677497 | SAMN19  677507 | SAMN19677510 | SAMN19677511 | SAMN19677512 |
| --- | --- | --- | --- | --- | --- | --- | --- | --- | --- |
| **Population ID** | | CM05101 | CC21A1 | AC21A1 | A00051 | CM05101 | CC21A1 | AC21A1 | A00051 |
| **Salinity** | | 0.8 M | 0.8 M | 0.8 M | 0.8 M | 4.0 M | 4.0 M | 4.0 M | 4.0 M |
| **Strain (CCAP)** | | 19/15 | 19/15 | 19/12 | 19/12 | 19/15 | 19/15 | 19/12 | 19/12 |
| **RNA-seq** | **Total PE reads** | 22.43 M | 20.84 M | 22.26 M | 21.30 M | 27.80 M | 27.73 M | 24.13 M | 25.24 M |
|  | **PE reads after trimming** | 22.30 M | 20.75 M | 22.17 M | 21.20 M | 27.68 M | 27.61 M | 24.03 M | 25.14 M |
|  | **%** | 99.42% | 99.59% | 99.59% | 99.55% | 99.55% | 99.59% | 99.62% | 99.62% |
|  | **Overall alignment rate** | 75.51% | 74.99% | 75.55% | 74.65% | 76.86% | 76.62% | 76.39% | 75.72% |
| **WGB-seq** | **Total PE reads** | 55.62 M | 54.45 M | 51.29 M | 55.04 M | 50.07 M | 43.79 M | 47.36 M | 47.71 M |
|  | **Estimated depth of coverage (×)** | 47.67 | 46.67 | 43.96 | 47.17 | 42.92 | 37.53 | 40.59 | 40.89 |
|  | **PE reads after trimming** | 55.32 M | 54.13 M | 50.71 M | 54.03 M | 48.91 M | 43.16 M | 46.39 M | 46.63 M |
|  | **%** | 99.46% | 99.43% | 98.88% | 98.17% | 97.68% | 98.58% | 97.95% | 97.74% |
|  | **Alignment with a unique best hit rate** | 36.01% | 35.97% | 31.85% | 31.44% | 35.66% | 35.47% | 29.56% | 30.32% |
|  | **Duplication rate** | 11.10% | 11.20% | 12.77% | 13.50% | 15.48% | 12.57% | 14.29% | 14.83% |
|  | **Unique alignements** | 17.71 M | 17.29 M | 14.09 M | 14.69 M | 14.74 M | 13.38 M | 11.75 M | 12.04 M |
|  | **Total C's** | 693.64 M | 662.21 M | 514.13 M | 522.66 M | 481.82 M | 466.74 M | 403.99 M | 403.19 M |
|  | **Methylated C's:** | | | | | | | | |
|  | **CpG context** | 45.23 M | 42.30 M | 33.27 M | 31.62 M | 25.15 M | 26.89 M | 23.97 M | 22.36 M |
|  | **CHG context** | 8.25 M | 9.07 M | 6.32 M | 5.28 M | 6.62 M | 6.63 M | 5.47 M | 4.60 M |
|  | **CHH context** | 5.61 M | 5.33 M | 4.38 M | 4.39 M | 4.25 M | 4.08 M | 3.48 M | 3.45 M |
|  | **Percentage methylation:** | | | | | | | | |
|  | **CpG context** | 51.33% | 49.84% | 51.01% | 46.29% | 38.24% | 43.71% | 46.09% | 41.22% |
|  | **CHG context** | 5.90% | 6.85% | 6.17% | 5.18% | 7.33% | 7.36% | 6.85% | 5.98% |
|  | **CHH context** | 1.20% | 1.20% | 1.26% | 1.25% | 1.31% | 1.29% | 1.28% | 1.27% |

**Table S2. Effects of salinity or initial density on population growth rate for CCAP 19/12 and 19/15.**

|  | **Estimate** | **Std. Error** | **Z value** | **Pr(>\|z\|)** |  |
| --- | --- | --- | --- | --- | --- |
| ***GLM #1_19/12: Initial population response (days 0 to 1) for Strain_19/12*** | | | | |  |
| Intercept (Strain_19/12 at Salinity 0.8 M and Day 0) | 9.865 | 0.039 | 252.519 | < 0.001 | ^***^ |
| Day:Salinity_0.8M | 0.264 | 0.097 | 2.715 | 0.007 | ^**^ |
| Day:Salinity_2.4M | 0.240 | 0.097 | 2.467 | 0.014 | ^*^ |
| Day:Salinity_4.0M | -1.537 | 0.098 | -15.760 | < 0.001 | ^***^ |
| ***GLM #1_19/15: Initial population response (days 0 to 1) for Strain_19/15*** | | | | |  |
| Intercept (Strain_19/15 at Salinity 0.8 M and Day 0) | 9.982 | 0.016 | 610.848 | < 0.001 | ^***^ |
| Day:Salinity_0.8M | 0.228 | 0.041 | 5.596 | < 0.001 | ^***^ |
| Day:Salinity_2.4M | 0.156 | 0.041 | 3.820 | < 0.001 | ^***^ |
| Day:Salinity_4.0M | -0.435 | 0.041 | -10.677 | < 0.001 | ^***^ |
| ***GLM #2_19/12: Population growth in exponential phase (days 1 to 4) for Strain_19/12*** | | | | | |
| Intercept (Strain_19/12 at Salinity 0.8 M and Day 1) | 10.317 | 0.087 | 118.434 | < 0.001 | ^***^ |
| Day | 0.541 | 0.047 | 11.612 | < 0.001 | ^***^ |
| Salinity_2.4M | -0.134 | 0.123 | -1.085 | 0.278 |  |
| Salinity_4.0M | -1.904 | 0.123 | -15.453 | < 0.001 | ^***^ |
| Day:Salinity_2.4M | 0.018 | 0.066 | 0.276 | 0.783 |  |
| Day:Salinity_4.0M | 0.095 | 0.066 | 1.448 | 0.148 |  |
| ***GLM #2_19/15: Population growth in exponential phase (days 1 to 4) for Strain_19/15*** | | | | | |
| Intercept (Strain_19/15 at Salinity 0.8 M and Day 1) | 10.374 | 0.049 | 212.419 | < 0.001 | ^***^ |
| Day | 0.596 | 0.026 | 22.850 | < 0.001 | ^***^ |
| Salinity_2.4M | -0.155 | 0.069 | -2.245 | 0.025 |  |
| Salinity_4.0M | -0.809 | 0.069 | -11.715 | < 0.001 | ^***^ |
| Day:Salinity_2.4M | -0.100 | 0.037 | -2.716 | 0.007 | ^**^ |
| Day:Salinity_4.0M | -0.316 | 0.037 | -8.549 | < 0.001 | ^***^ |
| ***GLM #3: Population growth in exponential phase (days 1 to 4) in salinity 4.0M*** | | | | |  |
| Intercept (Strain_19/12 initiated at N0 = 20,000 cells.mL^-1^, at Salinity 4.0 M and Day 1) | 8.413 | 0.087 | 97.236 | < 0.001 | ^***^ |
| Day | 0.636 | 0.046 | 13.758 | < 0.001 | ^***^ |
| Strain_19/15_N0_20,000 | 1.152 | 0.122 | 9.415 | < 0.001 | ^***^ |
| Strain_19/15_N0_5,000 | 0.150 | 0.141 | 1.063 | 0.288 | ^.^ |
| Day:Strain_19/15_N0_20,000 | -0.355 | 0.065 | -5.435 | < 0.001 | ^***^ |
| Day:Strain_19/15_N0_5,000 | -0.248 | 0.076 | -3.282 | < 0.001 | ^**^ |

General Linear Models (GLMs) with a negative binomial distribution were performed on cells count data for each of the two strains, where the interaction of time (Day) with salinity or strain estimates their effect on population exponential growth (or decline) rate. GLM#1 shows significant initial growth of both strains at salinities 0.8M and 2.4M, but significant initial decline at salinity 4M. GLM#2 shows that in the following days, strain CCAP 19/12 has similar growth rates at all salinities, while strain 19/15 growth faster at 0.8M. GLM#3 shows that at the highest salinity 4M, strain CCAP 19/15 grows significantly slower than strain 19/12, regardless of its initial density. This pattern may suggest that CCAP 19/12 experienced faster growth than CCAP 19/15 at high salinity because its initial decline resulted in reduced competition. Indeed CCAP 19/12 started growing again at a density of *c.* 4,500 cells.mL^-1^ in Day 1 (from GLM #2_19/12), while strain 19/15 did so around 14,250 cells.mL^-1^ (from GLM #2_19/15). However, even when CCAP 19/15 started at a much lower density of 5,000 instead of 20,000 cells.mL^-1^ on Day 0, is still had a significantly lower growth rate than 19/12 at salinity 4M (GLM #3).

**Table S3.** **Strain and salinity effects on population growth rate for isogenic populations.**

|  | **Estimate** | **Std. Error** | **z value** | **Pr(>\|z\|)** |  |
| --- | --- | --- | --- | --- | --- |
| ***GLM #1_Isogenic: Initial population response (days 0 to 1), starting from same initial density*** | | | | | |
| Intercept (Strain_19/12 at Salinity 0.8 M and Day 0) | 9.494 | 0.056 | 169.002 | < 2e-16 | ^***^ |
| Day | 0.813 | 0.173 | 4.697 | 0.000 | ^***^ |
| Day:Salinity_2.4M | -0.403 | 0.279 | -1.443 | 0.149 |  |
| Day:Salinity_4.0M | -3.586 | 0.228 | -15.696 | < 2e-16 | ^***^ |
| Day:Strain_19/15 | -0.198 | 0.228 | -0.870 | 0.384 |  |
| Day:Salinity_2.4M:Strain_19/15 | 0.058 | 0.395 | 0.147 | 0.883 |  |
| Day:Salinity_4.0M:Strain_19/15 | 3.045 | 0.323 | 9.438 | < 2e-16 | ^***^ |
| ***GLM #2_Isogenic: Population growth in exponential phase (days 1 to 4)*** | | | | |  |
| Intercept (Strain_19/12 at Salinity 0.8 M and Day 1) | 10.379 | 0.108 | 96.317 | < 2e-16 | ^***^ |
| Day | 0.577 | 0.058 | 10.024 | < 2e-16 | ^***^ |
| Salinity_2.4M | -0.411 | 0.187 | -2.202 | 0.028 | ^*^ |
| Salinity_4.0M | -3.252 | 0.153 | -21.288 | < 2e-16 | ^***^ |
| Strain_19/15 | -0.125 | 0.152 | -0.820 | 0.412 |  |
| Day:Salinity_2.4M | 0.028 | 0.100 | 0.279 | 0.780 |  |
| Day:Salinity_4.0M | 0.362 | 0.082 | 4.434 | 0.000 | ^***^ |
| Day:Strain_19/15 | 0.127 | 0.081 | 1.563 | 0.118 |  |
| Salinity_2.4M:Strain_19/15 | -0.091 | 0.264 | -0.343 | 0.731 |  |
| Salinity_4.0M:Strain_19/15 | 2.550 | 0.216 | 11.818 | < 2e-16 | ^***^ |
| Day:Salinity_2.4M:Strain_19/15 | -0.080 | 0.141 | -0.570 | 0.569 |  |
| Day:Salinity_4.0M:Strain_19/15 | -0.798 | 0.115 | -6.919 | 0.000 | ^***^ |

We founded four populations from single isolated cells for each of the two strains (following the protocol in Leung *et al.* 2020). As *D. salina* is haploid, a population founded from a single cell is expected to be isogenic. General Linear Models (GLMs) with a negative binomial distribution were performed on cells count data, where the interaction of time (Day) with salinity or strain estimates their effect on population exponential growth (or decline) rate, as in Table 1 in main text, but performed on isogenic populations.


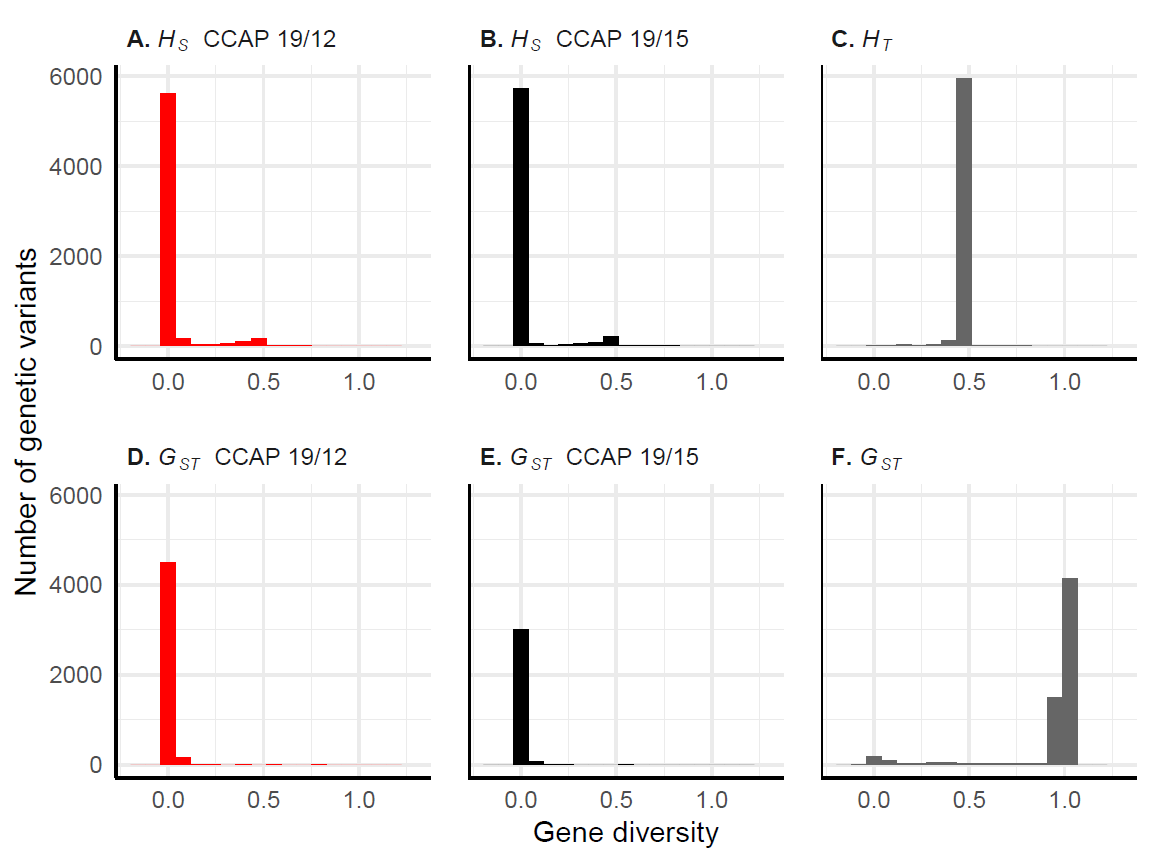


**Fig. S1. Genetic variation within and among populations.** Gene diversity within population (*H_S_*), averaged across populations of CCAP 19/12 (A) or 19/15 (B), total gene diversity across all populations (*H_T_*, C), and genetic differentiation (*G_ST_*) across populations of CCAP 19/12 (D), of 19/15 (E), or between strains (F).


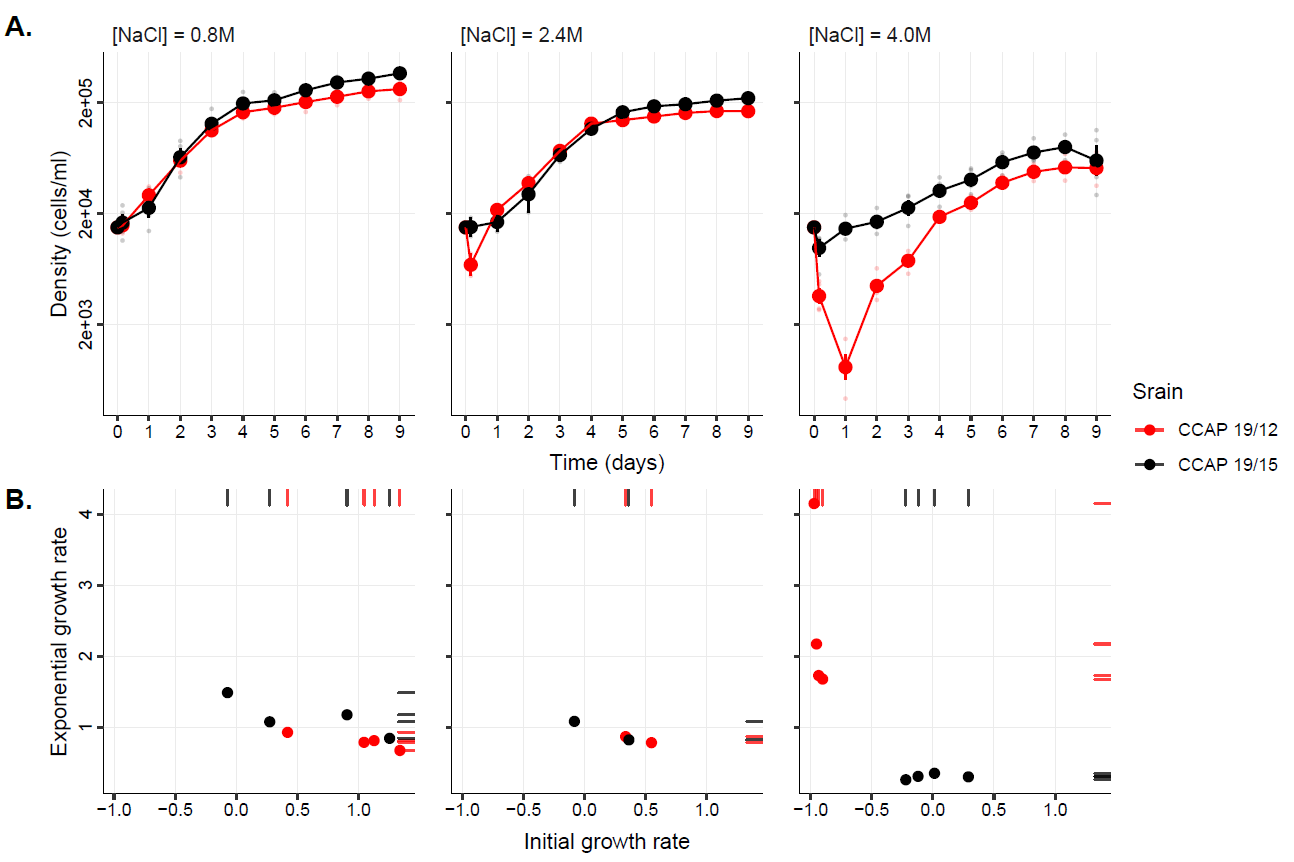


**Fig. S2. Dynamics of isogenic populations under different osmotic stresses.** Isogenic populations were founded from single isolated cells for each of the two strains following the protocol in Leung *et al.* (2020). As *D. salina* is haploid, a population founded from a single cell is expected to be isogenic. **A.** Mean population growth curves in three different salinities. For each strain (CCAP 19/12 and CCAP 19/15 in red and black, respectively), mean cell density and standard error were calculated from four isogenic populations (or two for the iso-osmotic condition). **B.** Exponential growth rate against initial growth or decline rate, under different osmotic regimes. Rug plots illustrate the distribution of the initial (days 0 to 1) and exponential (days 1 to 4) growth rates on their respective axes.


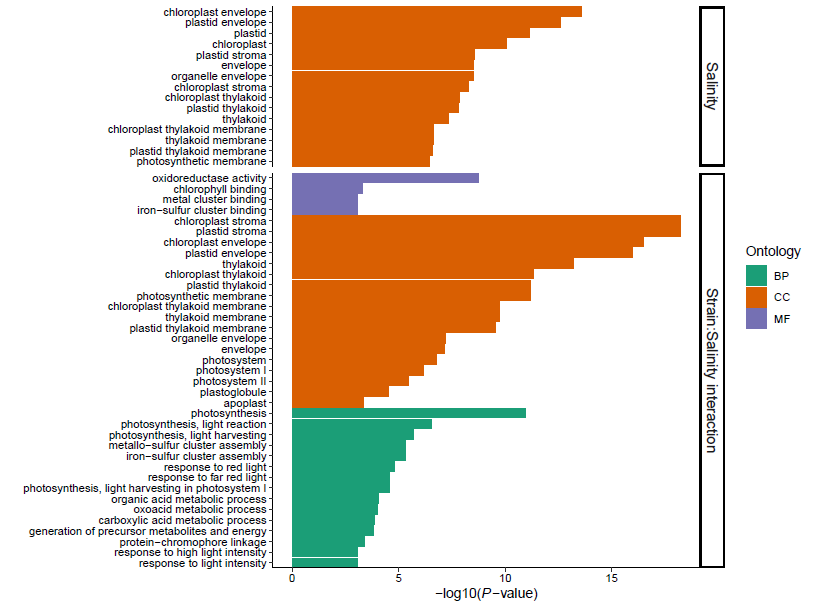


**Fig. S3. Gene ontology (GO) term enrichment analysis.** We adopted GO assignments to classify the functions of *D. salina* transcripts. Based on sequence homology to known sequence databases (BLAST+/SwissProt), a total of 9,874 *D. salina* transcripts (30.93% of 31,926 transcripts) were assigned at least one GO term and categorized into 12,543 GO terms. Enriched GO terms of the DE genes were then identified using Fisher’s exact test in the *topGO* R package (Alexa and Rahnenfuhrer, 2010). GO categories included molecular function (MF, purple), cellular component (CC, orange) and biological process (BP, green),) and were sorted by decreasing order of evidence within each category, based on the GO enrichment test *P*-value (for *P* ≤ 0.001) after Benjamini-Hochberg (BH) adjustment. No significant GO term enrichment was detected for DE transcripts between strains.

Alexa, A., & Rahnenfuhrer, J. (2010). topGO: enrichment analysis for gene ontology. *R package version*, *2*(0), 2010.
